# Career Advancement Challenges for Women in Tenure Versus Clinical Tracks in Academic Medicine: Cross-Sectional Survey Study

**DOI:** 10.2196/83374

**Published:** 2026-05-29

**Authors:** Susan Massick, Courtney Hebert, Sonja Chen, Shu-Hua Wang, Mengda Yu, Gloria P Fleming, Subhdeep Virk, Audrey M Sigmund, Kristy L Townsend, Maya S Iyer

**Affiliations:** 1 Department of Dermatology The Ohio State University College of Medicine Columbus, OH United States; 2 Department of Biomedical Informatics Division of Clinical Informatics and Implementation Science The Ohio State University College of Medicine Columbus, OH United States; 3 Department of Pathology The Ohio State University College of Medicine Columbus, OH United States; 4 Department of Pathology and Laboratory Medicine Nationwide Children's Hospital Columbus, OH United States; 5 Division of Infectious Diseases Department of Internal Medicine The Ohio State University College of Medicine Columbus, OH United States; 6 Center for Biostatistics The Ohio State University College of Medicine Columbus, OH United States; 7 Department of Ophthalmology The Ohio State University College of Medicine Columbus, OH United States; 8 Department of Psychiatry and Behavioral Health The Ohio State University College of Medicine Columbus, OH United States; 9 Division of Hematology Department of Internal Medicine The Ohio State University College of Medicine Columbus, OH United States; 10 Department of Neurosurgery The Ohio State University College of Medicine Columbus, OH United States; 11 Department of Pediatrics The Ohio State University College of Medicine Columbus, OH United States; 12 Department of Pediatrics Division of Emergency Medicine Nationwide Children's Hospital Columbus, OH United States

**Keywords:** academic promotion, career advancement, clinical track, tenure track, women-identifying faculty

## Abstract

**Background:**

Despite gender parity in medical school matriculants, women in academic medicine encounter ongoing challenges in career advancement and promotion.

**Objective:**

This study aimed to assess the experiences and challenges impacting the career trajectory for women-identifying faculty at the Ohio State University College of Medicine (OSUCOM) in tenure track (TT) and clinical track (CT; nontenure track) academic appointments.

**Methods:**

The OSUCOM Widening Impact in Medicine and Science (WIMS) organization, earlier known as Women in Medicine and Science, conducted a digital online survey in May 2023 distributed to all women-identifying faculty at OSUCOM. We focused on the responses of faculty who reported being on the TT or the CT at the rank of assistant, associate, or full professor. Survey data were compared to ongoing and new career development programming in WIMS that addresses faculty needs across both tracks.

**Results:**

Approximately half (639/1292, 49.5%) of all women-identifying OSUCOM faculty responded to the survey, with 565 (88.4%) reporting as TT or CT at the rank of assistant, associate, or full professor. Among these respondents, 23.5% (n=133) were on the TT (assistant professors: n=60, 45.1%; associate professors: n=37, 27.8%; professors: n=36, 27.1%), while 76.5% (n=432) were on the CT (assistant professors: n=245, 56.7%; associate professors: n=129, 29.9%; professors: n=58, 13.4%). Across both appointment tracks, of 492 participants, the two most common challenges impacting career advancement were caregiving responsibilities (n=271, 55.1%) and burnout (n=235, 47.8%). Significantly more CT versus TT faculty identified increased patient load (n=187, 47.8%, vs n=10, 9.9%; *P*<.001) and clinical productivity requirements (n=150, 38.4%, vs n=19, 18.8%; P=.002) as barriers to career advancement. CT faculty were also more likely to report not currently having a mentor compared to TT faculty (n=202, 48.9%, vs n=37, 31.9%; *P*=.001). In contrast, significantly more TT faculty reported the impact of COVID-19 (n=50, 49.5%, vs n=75, 19.2%; *P*<.001) and lack of funding (n=28, 27.7%, vs n=66, 16.9%; *P*=.014) as barriers to advancement. TT faculty were also significantly more likely to report sponsoring an OSU faculty member in their current position (n=55, 54.5%, vs n=147, 38.0%; *P*=.003).

**Conclusions:**

Our findings highlight that TT and CT women-identifying faculty experience different career landscapes with distinct track-specific barriers to professional advancement. CT women-identifying faculty are particularly vulnerable in areas such as mentorship and sponsorship, while TT women-identifying faculty experience more challenges related to research productivity. With an increasing number of women hired as CT faculty, it is critical for academic medical centers (AMCs) to maintain and strengthen support for all faculty types by broadening institutional-level strategies and offering targeted career development programming.

## Introduction

Women in academic medicine continue to experience challenges in career advancement, promotion, and leadership roles despite gender parity in medical school matriculation [1-3]. In 2023, women represented 52% of medical school graduates, 45% of full-time faculty, and 51% of full-time faculty under the age of 50 years, yet they represented less than one-third of professors and senior leaders (29% of professors, 25% of department chairs, and 27% of medical school deans) [4].

Since the 1980s, academic medical centers (AMCs) have undergone a substantial shift in faculty hiring away from tenure track (TT) to nontenure track clinical track (CT) appointments. More women-identifying faculty are being hired by AMCs, yet now the current trend is for both scientist and clinical faculty to be hired into nontenure academic tracks [5]. In 1982, the majority of full-time science faculty (78%) were hired into TT compared with nontenure appointments (17%). By 2022, TT hiring of full-time science faculty had decreased to 64%, while nontenure hiring had increased to 32% [6]. For faculty in clinical departments, full-time TT appointments dropped precipitously from 59% to 18% in the same time frame, with a corresponding rise in CT appointments. By 2022, 72% of full-time clinical faculty in AMCs with a tenure system were hired into CT appointments [6]. Currently, women faculty are disproportionately represented in CT positions and less likely to be appointed to TT positions than men [5]. These hiring trends may have an ongoing impact on career advancement and promotion with the increasing emphasis that AMCs place on clinical productivity and clinical revenue over traditional scholarly productivity [4,5,7]. The current literature indicates higher rates of promotion for those along TT over CT positions [8], yet there are limited data examining the potential impact of structural differences across appointment tracks.

At the Ohio State University College of Medicine (OSUCOM), TT faculty are evaluated on research and scholarly contributions. TT faculty have protected research time but must meet promotion criteria within a certain period to attain tenure. CT faculty primarily engage in clinical care and do not have time clock requirements for promotion but also generally have less protected research time [9]. Consistent with national trends, OSUCOM has had a significant increase in CT appointments. From 2008 to 2025, among all academic ranks at OSUCOM, there has been a consistent decline in the proportion of TT appointments from 55.5% to 22.1%, with a steady increase in CT appointments from 44% to 76% [10].

Web-based survey platforms allow data collection of faculty experiences across ranks and tracks. The OSUCOM Widening Impact in Medicine and Science (WIMS) organization, earlier known as Women in Medicine and Science, is committed to evidence-based, data-driven programming for career development. In 2023, WIMS created and disseminated a college-wide online survey to evaluate the experiences of women-identifying faculty and challenges impacting the career trajectory and advancement. This paper outlines the initial findings of this survey, highlighting broader trends and ongoing challenges for women-identifying faculty at a single large AMC. The study presents shared challenges and reveals significant differences in experiences and career advancement needs of TT and CT faculty.

## Methods

### Study Design

The WIMS Advocacy Committee developed and disseminated a cross-sectional digital survey (Qualtrics, Provo, Utah) to all women-identifying faculty at OSUCOM in 2023. Survey questions were informed by established thematic domains from the faculty advancement literature. To ensure understanding of survey questions, the survey was pilot-tested among a small group of WIMS members, selected OSUCOM faculty, and by the OSUCOM’s Office of Faculty Affairs.

Eligible individuals included any women-identifying faculty across all ranks and departments at OSUCOM. Invitation emails with a link to participate in the online Qualtrics survey were distributed to all eligible individuals, with two reminder emails to complete the survey while the survey link was active from April 11 to May 23, 2023.

### Data Collection

Survey questions (Multimedia Appendix 1) focused on demographics, including academic rank, appointment track, race, ethnicity, mentorship, and sponsorship. These topic domains were selected to assess workplace trends, individual experiences, and faculty perspectives.

We obtained historical data on demographics (gender identification, track, and rank) from OSUCOM’s Office of Faculty Affairs to better understand the context of survey responses and to assess the degree of participation in the survey. At OSUCOM, there are four faculty academic appointment tracks: tenure, clinical, research, and associated. The academic ranks are designated as instructor, assistant professor, associate professor, and professor and are available on all four tracks. Although the survey allowed for any combination of responses, for this study, our primary analysis focused on faculty participants on the TT or the CT who were assistant, associate, or full professor. We excluded responses from instructors as well as research and associated track respondents.

### Statistical Analysis

We used descriptive statistics to analyze and summarize responses by TT and CT using means (SDs) for continuous variables and frequencies and percentages for categorical variables. These characteristics were compared across tracks using chi-square tests for categorical variables and *t* tests for continuous variables. Effect size calculations were not included in this descriptive exploratory analysis. We assessed the representativeness of our sample compared to the population by comparing key demographics. Given our focus on inference within subgroups rather than population-level estimation, we chose descriptive comparisons rather than using statistical tests to compare. Although internal consistency statistics were not calculated, we analyzed qualitative data using content and thematic analysis for open-ended questions.

### Ethical Considerations

This study was reviewed and determined to be exempt by the Ohio State University Institutional Review Board (IRB study ID 2022E1238) under Qualifying Exempt Category #2b for minimal risk research involving survey procedures. The study involved a voluntary online survey of faculty participants in which no identifiable personal information was collected, and all responses were recorded in a manner that preserved participant confidentiality. An informed consent statement through the recruitment email explicitly stated the study’s objectives, that study participation was voluntary, and that clicking the link to open the survey constituted informed consent to participate. To further preserve confidentiality of faculty, we suppressed cell counts<10 in the results. Responses were anonymous, and participants could choose to partially or fully complete the survey, as well as discontinue at any point in the survey. Although no direct compensation was provided, participants had the option to voluntarily enter a separate anonymous raffle for one of eight US $50 Amazon gift cards as an incentive. Raffle entries were collected independently to preserve participant anonymity. All study procedures adhered to institutional policies and ethical standards for research involving human subjects.

## Results

### Faculty Details

This study revealed significant differences in experiences and career advancement needs of TT and CT faculty. In 2023, OSUCOM had 1292 women-identifying faculty, of which 49.5% (n=639) responded to the survey. Our total sample for this study included 565 (88.4%) women-identifying faculty who were appointed to the TT (n=133, 23.5%) and the CT (n=432, 76.5%) at the rank of assistant professor (n=305, 54.0%), associate professor (n=166, 29.4%), and professor (n=94, 16.7%), as shown in Table 1. These proportions are similar to concurrent data from OSUCOM’s Office of Faculty Affairs showing that among all women-identifying faculty at OSUCOM in 2023, 16.6% were TT faculty and 81.2% were CT faculty, with 65.2% assistant professors, 21.9% associate professors, and 12.8% professors across both the TT and the CT [10].

**Table 1:** Descriptive summary by promotion track.

Variable and level		TT^a^ faculty (n=133), n (%)	CT^b^ faculty (n=432), n (%)	Total faculty (N=565), n (%)	*P* value
**Completed survey**					
	Yes	100 (75.2)	386 (89.4)	486 (86.0)	<.001
	No	33 (24.8)	46 (10.7)	79 (14.0)	—^c^
**Current rank**					
	Assistant professor	60 (45.1)	245 (56.7)	305 (54.0)	<.001
	Associate professor	37 (27.8)	129 (29.9)	166 (29.4)	—
	Professor	36 (27.1)	58 (13.4)	94 (16.6)	—
**Years in current rank**					
	0-5	93 (70.5)	297 (69.1)	390 (69.4)	<.001
	6-10	17 (12.8)	102 (23.7)	119 (21.2)	—
	≥11	22 (16.7)	31 (7.2)	53 (9.4)	—
	Missing^d^	1 (0.8)	2 (0.5)	3 (0.5)	—
**Race**					
	White	91 (75.2)	280 (67.2)	371 (69.0)	.624
	Asian	14 (11.6)	70 (16.8)	84 (15.6)	—
	Black/African American	5 (4.1)	17 (4.1)	22 (4.1)	—
	Multiracial	3 (2.5)	18 (4.3)	21 (3.9)	—
	Other	3 (2.5)	13 (3.1)	16 (3.0)	—
	Prefer not to answer	5 (4.1)	19 (4.6)	24 (4.5)	—
	Missing^d^	12 (9.0)	15 (3.5)	27 (4.8)	—
**Ethnicity**					
	Hispanic/Latino	8 (6.6)	24 (5.8)	32 (6.0)	.690
	Non-Hispanic/Latino	105 (86.8)	370 (89.4)	475 (88.8)	—
	Prefer not to answer	8 (6.6)	20 (4.8)	28 (5.2)	—
	Missing^d^	12 (9.0)	18 (4.2)	30 (5.3)	—
**Discipline**					
	Medical subspecialty^e^	80 (64.0)	337 (80.4)	417 (76.7)	<.001
	Surgical subspecialty^f^	13 (10.4)	59 (14.1)	72 (13.2)	—
	Basic science^g^	17 (13.6)	9 (2.2)	26 (4.8)	—
	Prefer not to answer	15 (12.0)	14 (3.3)	29 (5.3)	—
	Missing^d^	8 (6.0)	13 (3.0)	21 (3.7)	—

^a^TT: tenure track.

^b^CT: clinical track.

^c^Not applicable.

^d^“Missing” is a count of the number of people in the included cohort who did not provide an answer to that specific question. The percentages calculated for each variable are out of those who did answer.

^e^Medical subspecialties include dermatology, emergency medicine, family and community medicine, internal medicine, neurology, pathology, pediatrics, physical medicine and rehabilitation, psychiatry and behavioral health, radiation oncology, School of Health and Rehabilitation Science, and radiology.

^f^Surgical subspecialties include anesthesiology, obstetrics/gynecology, ophthalmology, orthopedics, ear-nose-throat, plastic and reconstructive surgery, surgery, and urology.

^g^Basic sciences include biological chemistry, biomedical education and anatomy, biomedical informatics, cancer biology, microbial infection and immunity, neuroscience, physiology, and cell biology.

Most respondents (n=390, 69.4%) had been in their current rank for 0-5 years. Years in rank differed between TT and CT faculty: more participants in the TT reported ≥11 years in rank (n=22, 16.7%) versus the CT (n=31, 7.2%), and more CT reported 6-10 years in rank (n=102, 23.7%) versus the TT (n=17, 12.8%; *P*=.001). Among professors, 23.7% (22/94) had been in their current rank for ≥11 years compared to 4.6% (14/305) of assistant professors (*P*<.001).

OSUCOM has 28 departments and 1 school. In this study, 25 departments and 1 school were represented. The highest number of respondents came from the Departments of Pediatrics (n=142, 22.2%) and Internal Medicine (n=118, 18.5%), which are the two largest departments at OSUCOM. We aggregated departments into medical, surgical, and basic science departments for ease of comparison. Medical and surgical subspecialties had a higher percentage of CT faculty compared to TT faculty (medical: n=337, 80.4%, vs n=80, 64.0%; surgical: n=59, 14.1%, vs n=13, 10.4%; *P*<.001). TT faculty made up the majority of basic science subspecialties (n=17, 65.4%).

### Mentorship: Quantitative Results

Table 2 summarizes mentorship quantitative results. Lack of mentorship was noted by 48.9% (n=202) of CT faculty compared to 31.9% (n=37) of TT faculty (*P*=.001). Lack of mentorship also differed significantly by rank, with 65.9% (n=56) of professors, 46.7% (n=71) of associate professors, and 38.4% (n=112) of assistant professors reporting not currently having a mentor (*P*<.001). Among assistant professors, 45.1% (107/237) of CT faculty reported no formal mentor compared to 9.1% (5/55) of TT faculty (*P*<.001).

**Table 2:** Mentorship, sponsorship, appointment, and promotion responses by track.

Variable and level			TT^a^ faculty (n=133), n (%)		CT^b^ faculty (n=432), n (%)		Total faculty (N=565), n (%)		*P* value
**Total faculty with mentors**									
	Yes	79 (68.1)		211 (51.1)		290 (54.8)		.001	
	No	37 (31.9)		202 (48.9)		239 (45.2)		—^c^	
	Missing	17 (12.8)		19 (4.4)		36 (6.4)		—	
**Having a mentor by rank**									
	Yes (assistant professor)	50 (43.1)		130 (31.5)		180 (34.0)		<.001	
	No (assistant professor)	5 (4.3)		107 (25.9)		112 (21.2)		—	
	Yes (associate professor)	15 (12.9)		66 (16.0)		81 (15.3)		.540	
	No (associate professor)	16 (13.8)		55 (13.3)		71 (13.4)		—	
	Yes (professor)	14 (12.1)		15 (3.6)		29 (5.5)		.072	
	No (professor)	16 (13.8)		40 (9.7)		56 (10.6)		—	
	Missing	17 (12.8)		19 (4.4)		36 (6.4)		—	
**Serving as a mentor**									
	Yes	70 (64.8)		194 (48.3)		264 (51.8)		.002	
	No	38 (35.2)		208 (51.7)		246 (48.2)		—	
	Missing	25 (18.8)		30 (6.9)		55 (9.7)		—	
**How is a mentor determined?**									
	Self-initiated	70 (88.6)		180 (86.5)		250 (87.1)		.640	
	Assigned	9 (11.4)		28 (13.5)		37 (12.9)		—	
	Missing	54 (40.6)		224 (51.9)		278 (49.2)		—	
**Focus of mentoring relationship**									
	Career development	30 (39.0)		111 (53.9)		141 (49.8)		<.001	
	Research/scholarship	33 (42.9)		34 (16.5)		67 (23.7)		—	
	Promotion and tenure	8 (10.4)		19 (9.2)		27 (9.5)		—	
	Clinical service	0 (0.0)		11 (5.3)		11 (3.9)		—	
	Work-life balance	1 (1.3)		8 (3.9)		9 (3.2)		—	
	Teaching	0 (0.0)		8 (3.9)		8 (2.8)		—	
	Administrative	2 (2.6)		5 (2.4)		7 (2.5)		—	
	Other	3 (3.9)		10 (4.9)		13 (4.6)		—	
	Missing	56 (42.1)		226 (52.3)		282 (49.9)		—	
**Do you feel that you have adequate sponsorship from senior faculty at OSU^d^ to achieve national recognition?**									
	Yes	45 (45.0)		143 (36.7)		188 (38.4)		.126	
	No	55 (55.0)		247 (63.3)		302 (61.6)		—	
	Missing	33 (24.8)		42 (9.7)		75 (13.3)		—	
**In your current position, have you sponsored an OSU faculty member?**									
	Yes	55 (54.5)		147 (38.0)		202 (41.4)		.003	
	No	46 (45.5)		240 (62.0)		286 (58.6)		—	
	Missing	32 (24.1)		45 (10.4)		77 (13.6)		—	

^a^TT: tenure track.

^b^CT: clinical track.

^c^Not applicable.

^d^OSU: Ohio State University.

A lower proportion of CT faculty compared to TT faculty reported serving as a mentor in their department (n=194, 48.3%, vs n=70, 64.8%; *P*=.002). Table 3 illustrates the differences in mentoring needs by academic track. Both TT and CT faculty desired mentorship in negotiation (n=206, 41.9%), leadership (n=230, 46.8%), and understanding and leveraging available OSUCOM resources (n=284, 57.7%). CT faculty were also significantly more likely than TT faculty to report wanting more mentorship in collaboration (n=148, 37.9%, vs n=19, 18.8%; *P*<.001) and in work-life balance (n=138, 35.3%, vs n=22, 21.8%; *P*=.01).

**Table 3:** Topics identified for increased mentorship among participants by academic track.

Topic and response		TT^a^ faculty (n=101), n (%)	CT^b^ faculty (n=391), n (%)	Total faculty (N=492), n (%)	*P* value
**Collaborations: initiating and maintaining**					
	Yes	19 (18.8)	148 (37.9)	167 (33.9)	<.001
	No	82 (81.2)	243 (62.1)	325 (66.1)	—^c^
**Communicating with individuals with different learning styles**					
	Yes	8 (7.9)	55 (14.1)	63 (12.8)	.099
	No	93 (92.1)	336 (85.9)	429 (87.2)	—
**Educating trainees**					
	Yes	17 (16.8)	85 (21.7)	102 (20.7)	.278
	No	84 (83.2)	306 (78.3)	390 (79.3)	—
**Grant writing**					
	Yes	26 (25.7)	82 (21.0)	108 (22.0)	.302
	No	75 (74.3)	309 (79.0)	384 (78.1)	—
**Leadership skills**					
	Yes	55 (54.5)	175 (44.8)	230 (46.8)	.082
	No	46 (45.5)	216 (55.2)	262 (53.3)	—
**Multidisciplinary teamwork**					
	Yes	19 (18.8)	71 (18.2)	90 (18.3)	.880
	No	82 (81.2)	320 (81.8)	402 (81.7)	—
**Negotiation**					
	Yes	43 (42.6)	163 (41.7)	206 (41.9)	.872
	No	58 (57.4)	228 (58.3)	286 (58.1)	—
**Other**					
	Yes	18 (17.8)	49 (12.5)	67 (13.6)	.167
	No	83 (82.2)	342 (87.5)	425 (86.4)	—
**Understanding and leveraging available resources in OSUCOM^d^/AMC^e^**					
	Yes	52 (51.5)	232 (59.3)	284 (57.7)	.155
	No	49 (48.5)	159 (40.7)	208 (42.3)	—
**Work-life balance**					
	Yes	22 (21.8)	138 (35.3)	160 (32.5)	.010
	No	79 (78.2)	253 (64.7)	332 (67.5)	—

^a^TT: tenure track.

^b^CT: clinical track.

^c^Not applicable.

^d^OSUCOM: Ohio State University College of Medicine.

^e^AMC: academic medical center.

### Mentorship: Qualitative Results

Thematic analysis of open-ended responses illustrated recurrent themes contributing to a lack of mentorship (Table 4). A total of 139 (24.6%) participants provided an open-ended response where common themes emerged. Participants noted a lack of knowledge and time regarding mentorship, a lack of time to develop relationships, a lack of formal assignment of a mentor, and a lack of departmental advocacy for a formal mentorship process. These concerns were described by 44.6% (n=62) of assistant professors, 21.6% (n=30) of associate professors, and 7.2% (n=10) of professors. A limited pool of available and formal institutional mentors was described by 14.4% (n=20) of assistant professors, 18.7% (n=26) of associate professors, and 5.0% (n=7) of professors. Loss of a mentor or having an external informal mentor was reported by 17.3% (n=24) of assistant professors, 15.8% (n=22) of associate professors, and 12.2% (n=17) of professors. Respondents also reported feeling that they did not need a mentor once they were promoted or achieved their personal career fulfillment.

**Table 4:** Reasons for lack of mentorship, lack of sponsorship, and career barriers.

Theme and reason			Representative quotes
**Reasons for lack of mentorship**			
	Lack of knowledge on how to obtain mentorship	“I have not been assigned a mentor and did not realize this was an option.” [Participant #272, assistant professor, TT^a^] “I was never assigned one or made aware of how to secure one when I started as an assistant professor.” [Participant #263, professor, TT] “No one has been assigned to me, and/or it has not been a priority to get faculty to connect on any regular level in person to meet each other and find out who might fit the bill.” [Participant #587, assistant professor, CT^b^]	
	Lack of available mentors at the institution	“Very hard to find one. As a professor, everyone thinks you should be fine, you have made it. That is simply not true. I still need guidance particularly when it comes to navigating resources at OSU^c^ and continuing to develop my science.” [Participant #80, professor, TT] “There do not seem to be many ‘elder’ physicians interested in mentoring (did the ‘mentor match’ process years ago and there were no available matches.” [Participant #138, associate professor, CT] “Very few available for individuals at the professor level.” [Participant #362, professor, CT]	
	Mentor retired/left the institution	“They all left OSU; my mentors are outside OSU now.” [Participant #194, associate professor, TT] “My research mentor left OSU, and I have not found another. I do not have anyone senior in my department in my exact specialization, so it has been hard to find someone.” [Participant #338, associate professor, CT] “Both mentors retired or left institution and did not replace.” [Participant #508, professor, CT]	
	Do not feel the need for a mentor	“Although I have had a mentor earlier in my career, I have enough experience now that it does not seem necessary.” [Participant #339, associate Professor, TT] “Promoted in last few years, and I have not been as proactive in continuing to seek mentorship.” [Participant #185, associate professor, CT] “Senior enough to not want a mentor or need a mentor.” [Participant #216, professor, CT]	
**Reasons for lack of sponsorship**			
	Lack of institutional/departmental structure to offer sponsorship	“Lack of senior leaders in my field at OSU.” [Participant #260, assistant professor, TT] “Our previous division director was an excellent sponsor. Our current division director, while very supportive, seems to do less active sponsoring.” [Participant #582, associate professor, CT] “I am the only person of my specialty on my team, so sponsorship opportunities are sparse. Sponsorship opportunities are sparse for [my field], in general, at the College of Medicine.” [Participant #599, associate professor, CT]	
	Lack of knowledge on how to obtain sponsorship	“I was not even familiar with this term before this survey.” [Participant #16, assistant professor, CT] “Do not know how to seek this out.” [Participant #87, assistant professor, CT]	
	Lack of available sponsors at the Institution	“All of my sponsorship has been done by colleagues outside of [the institution].” [Participant #93, associate professor, TT] “I have been sponsored by many outside of the institution, but it has been limited within the institution.” [Participant #52, assistant professor, CT]	
**Career barriers**			
	Lack of leadership support	“Poor Vice Chair leadership; [spouse] having abusive mentor in basic science that led to unequal ability to balance parenting.” [Participant #111, assistant professor, TT] “Culture, lack of advocacy by more senior people.” [Participant #39, associate professor, TT] “Division director not supportive.” [Participant #329, assistant professor, CT] “I believe it should have been recommended for me to go up for promotion both to associate and to full professor earlier. No one advised me to do so. I had to independently start the process myself.” [Participant #423, associate professor, CT]	
	Effort not recognized for promotion	“Unequal distribution of teaching responsibilities and zero departmental equity.” [Participant #496, associate professor, TT] “I do too much in education but not enough to be promoted via that pathway. Most of what I do outside of education is more clinical, but I do not want to give up the education piece…it just feels too overwhelming and not worth it at this point now.” [Participant #609, assistant professor, CT] “I have worked in the military and non-academic settings—these work experiences are not considered in my rank.” [Participant #104, assistant professor, CT] “My education responsibilities do not count toward my productivity, increasing overall workload.” [Participant #79, assistant professor, CT]	
	Staff shortage	“The COVID impact is less that things shut down in 2020 and more that things are still broken now. Everywhere is short-staffed, so there is less help. Everyone is burnt out, so there is fewer gung-ho students/residents to take on side projects. COVID was hard, but the lack of recovery to pre-COVID has been harder.” [Participant #414, assistant professor, TT] “It is difficult to recruit personnel.” [Participant #273, professor, TT] “Significantly increased clinical load due to lot of other staff leaving OSU (nurses, case managers, pharmacists) so a lot of the dedicated research time is [used] for clinical work instead.” [Participant #291, assistant professor, CT]	
	Lack of understanding of the promotion process	“The promotion application itself is ridiculously burdensome. More than 50 hours of work just to apply for promotion.” [Participant #201, assistant professor, CT] “Specifically, I was not assigned a P&T mentor until I was in my [thi]rd year and was not made aware of the specific P&T requirements. The department itself was disorganized in tracking junior faculty (unless they advocated for themselves) and missed my [four]th year review. This has set me back a full year on my tenure track.” [Participant #403, assistant professor, TT]	

^a^TT: tenure track.

^b^CT: clinical track.

^c^OSU: Ohio State University.

### Sponsorship: Quantitative Results

More than half of both TT and CT faculty reported a lack of adequate sponsorship. CT faculty were less likely to report sponsoring another faculty member in their current position compared to TT faculty (n=147, 38.0%, vs n=55, 54.5%; *P*=.003). Opportunities for sponsorship differed between TT and CT (Table 5). Compared to CT faculty, TT faculty were significantly more likely to report receiving sponsorship for activities such as invitations to join an editorial board, serve as a grant reviewer, be appointed to institutional search committees, serve on a national conference panelist, or be nominated for a national society committee.

**Table 5:** Sponsorship opportunities participants received.

Opportunity	TT^a^ faculty (n=101), n (%)	CT^b^ faculty (n=391), n (%)	Total faculty (N=492), n (%)	*P* value
First/senior authorship	12 (11.9)	57 (14.6)	69 (14.0)	.487
Editorial board	16 (15.8)	20 (5.1)	36 (7.3)	<.001
Grand rounds	17 (16.8)	61 (15.6)	78 (15.9)	.763
Grant reviewer	24 (23.8)	11 (2.8)	35 (7.1)	<.001
Institutional committee work	28 (27.7)	76 (19.4)	104 (21.1)	.069
Institutional search committee(s)	20 (19.8)	21 (5.4)	41 (8.3)	<.001
Leadership development programs	23 (22.8)	82 (21.0)	105 (21.3)	.694
National conference presentation(s)	23 (22.8)	71 (18.2)	94 (19.1)	.293
National conference panelist/moderator	24 (23.8)	54 (13.8)	78 (15.9)	.015
National society committee nominations	27 (26.7)	54 (13.8)	81 (16.5)	.002
Nomination for awards/recognition	24 (23.8)	63 (16.1)	87 (17.7)	.072
Nomination to mission-critical or high-visibility roles in division/department	11 (10.9)	46 (11.8)	57 (11.6)	.807
Publications	13 (12.9)	59 (15.1)	72 (14.6)	.574
Social media recognition	7 (6.9)	17 (4.4)	24 (4.9)	.283
Other	4 (4.0)	25 (6.4)	29 (5.9)	.355

^a^TT: tenure track.

^b^CT: clinical track.

### Sponsorship: Qualitative Results

A total of 64 participants provided comments regarding sponsorship. Prominent themes are represented in Table 4, including descriptions of being more often sponsored by external sources rather than internally/institutionally (n=15, 23.4%), a lack of institutional/departmental structure in creating opportunities for sponsorship (n=14, 21.9%), and being unaware of the concept of sponsorship and how to attain it (n=6, 9.4%).

### Career Advancement Barriers: Quantitative Results

**Figure 1:**
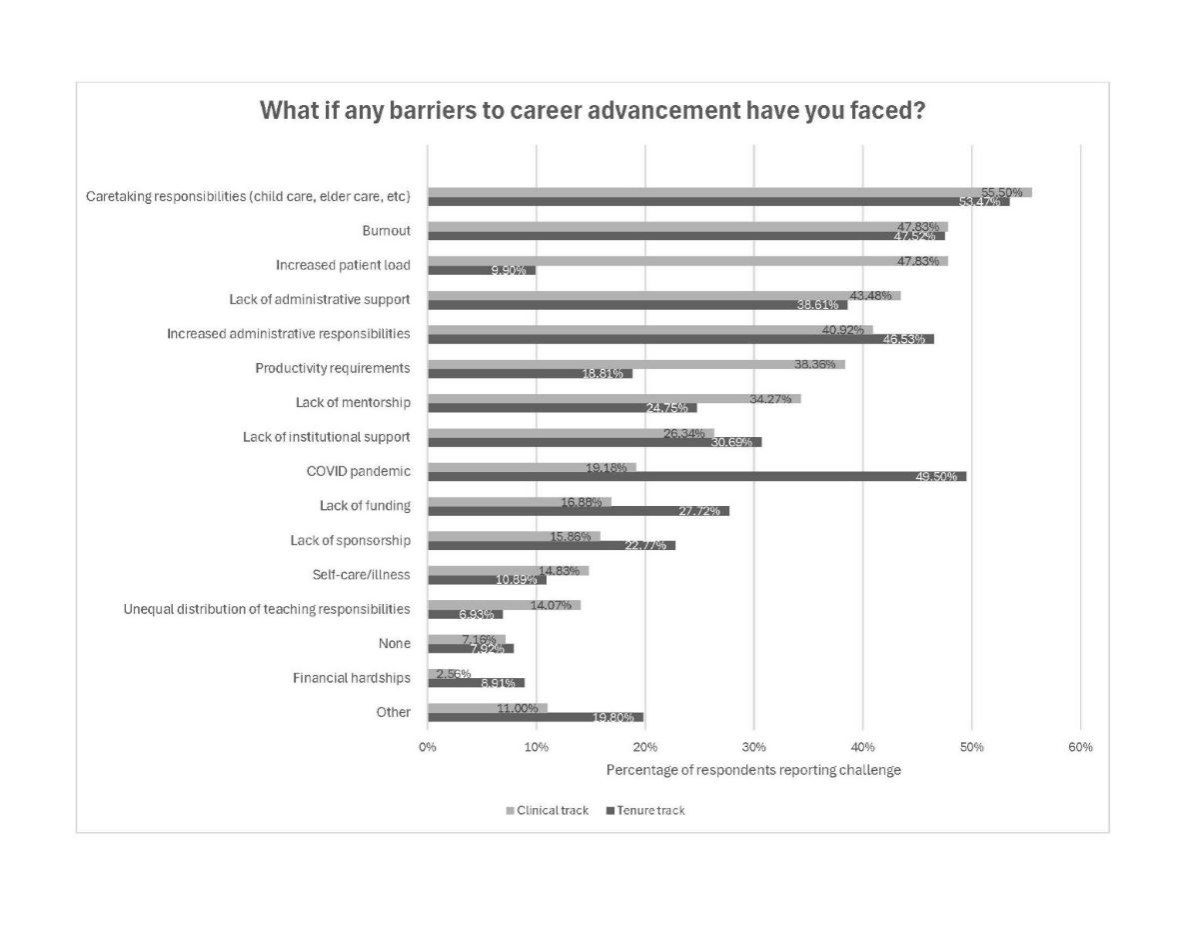
Career advancement barriers by track among women-identifying faculty survey participants at the Ohio State University College of Medicine. See high resolution version in Multimedia Appendix 3.

A total of 492 participants responded to the question, “What, if any, barriers to career advancement have impacted your career path?” (Figure 1 and Multimedia Appendix 2). The median number of barriers reported was 4 (IQR 3-6, range 0-11). Across all academic appointment tracks and faculty ranks, the most identified barriers to career advancement were caregiving (n=271, 55.1%), burnout (n=235, 47.8%), a lack of administrative support (n=209, 42.5%), and increased administrative responsibilities (n= 207, 42.1%), as shown in Figure 1. Significantly more CT faculty compared to TT faculty reported increased patient load (n=187, 47.8%, vs n=10, 9.9%; *P*<.001) and clinical productivity requirements (n=150, 38.4%, vs n=19, 18.8%; *P*=.002) as career advancement barriers. Conversely, TT faculty were more likely to report the impact of the COVID-19 pandemic (n=50, 49.5%, vs n=75, 19.2%; *P*<.001) and lack of funding (n=28, 27.7%, vs n=66, 16.9%; *P*=.014) as barriers.

### Career Barriers: Qualitative Results

A total of 75 participants who selected “Other” as a response to the survey question pertaining to career barriers provided additional open-ended comments further describing barriers to career advancement (Table 4). Major themes included a lack of administrative or departmental support (n=11, 14.7%), efforts not recognized as metrics for promotion (n=10, 13.3%), staff shortages in clinical or research areas (n=8, 10.7%), and challenges and confusion navigating the promotion process (n=8, 10.7%).

## Discussion

### Principal Findings

This study examined the findings of a cross-sectional, web-based survey of women-identifying faculty at a large US AMC. Our data corroborate national trends for AMCs showing a decline in TT appointments amid a rising number of CT appointments [5,6]. At OSUCOM, 81.2% of all women-identifying faculty are now on the CT [10]. Women-identifying faculty across both the TT and the CT in our survey shared career advancement barriers of caregiving responsibilities, burnout, and increased administrative duties with lower administrative support. More notably, our findings also highlight significant track-specific distinctions, thus illustrating differing needs among academic appointment tracks. These diverging challenges may also reveal broader structural inequities in academic medicine regarding academic identity, institutional support, and career opportunity [5,6].

Prior research has shown that TT appointments are associated with higher rates of promotion compared to CT appointments regardless of rank or department [8]. Assistant professors in clinical departments are promoted at lower rates than in basic science departments, and CT faculty overall are promoted at lower rates compared to TT faculty [8]. TT faculty have a formal time clock to attain tenure, with either termination or appointment/reassignment to the CT if they do not meet promotion metrics within the probationary period [6,11]. Although TT advancement is measured by traditional scholarly activity, such as grant funding, prominent research talks (national/international reputation), and publications, CT roles are evaluated and measured by clinical productivity [4,5,7]. Clinical contributions, such as high-quality patient care, clinical care innovations, and institutional service, may be underrecognized and undervalued as promotion criteria at AMCs [12].

Women faculty, regardless of the academic track, often carry a heavier burden of caregiving responsibilities, yet they also bear a disproportionate load of the education and service responsibilities that take time away from research time or scholarly productivity [5,13]. These barriers are amplified for those with intersectional identities, and these intersectional identities, although not evaluated in this study, may also impact hiring into certain academic tracks. Faculty in our study noted the impact of caregiving responsibilities on their career development. Teaching and service activities do not always factor significantly toward scholarly contributions that are valued for promotion [5]. Furthermore, across ranks, women, compared to men, are less likely to receive grant funding, speaker opportunities, or career development awards [5,12]. As a result of clinical care responsibilities and limited protected time for scholarship, CT faculty frequently have lower scholarly productivity, fewer leadership opportunities, and higher attrition compared with TT faculty [14-17]. As AMCs now shift hiring practices from TT to CT appointments and hire more women into CT roles to meet clinical service demands and clinical revenue needs [5,8,14,18,19], career development programming must be thoughtfully implemented in order to support the careers of all faculty across ranks [8]. In response to survey results and noted trends, OSUCOM has taken steps to implement strategies and targeted career development programming at the institutional level to benefit all faculty.

#### Mentorship Programming

Mentorship remains essential throughout all stages of academic progression and supports personal growth, professional development, and academic career advancement [20,21]. Mentorship is strongly associated with increased research productivity, job satisfaction, and faculty retention and promotion [21-23]. Mentorship plays a critical role in faculty development yet may not be equally available across appointment tracks.

Our findings show that the mentorship gap, particularly within CT roles, widens as faculty progress from assistant professor to a more senior rank. There was a notable drop in mentorship at the rank of professor, with 65.9% (n=56) participants noting a lack of mentorship. One possible explanation is that faculty transition from mentee to mentor as they progress through rank or no longer require traditional mentor support as they did earlier in their careers (Table 4). Although most new hires at AMCs are currently CT appointments, the CT is still a relatively new track in academic medicine with fewer CT professors compared to TT professors, fewer senior mentors available, and less formalized mentorship infrastructure than in the established TT [5,20]. Institutional interventions using organizational strategies toward structural change have also shown measurable positive outcomes, particularly as they pertain to mentorship, leadership development, and faculty retention [7,24]. Therefore, formal mentoring structures would benefit all faculty by expanding formalized CT mentorship and maintaining established TT mentorship.

At OSUCOM, WIMS continues to develop career development programming to address the mentorship and sponsorship needs identified by both TT and CT faculty. WIMS works closely with the Center for Faculty Advancement, Mentoring, and Engagement (FAME) and promotes the use of OSUCOM’s *Mentorcliq* platform, a formalized online program that matches mentors and mentees based on professional, academic, leadership, and scholarly career interests [25]. The WIMS Outreach Committee is developing a mentorship program with the Women in Medicine (WIM) medical student organization and Graduate Women in Science (GWIS) to provide further mentorship opportunities for the next generation of physicians and scientists. Networking opportunities at WIMS events also provide informal settings for faculty to meet and organically develop new mentoring relationships, particularly with other women-identifying or underrepresented gender faculty who experience similar career hurdles. Finally, survey results have been shared with OSUCOM leadership, including the department chairs/division chiefs, to ensure accountability and develop plans for change.

#### Sponsorship Programming

Distinct from mentorship, sponsorship also plays a critical role in faculty development. The three critical components of sponsorship include (1) focus on career advancement, (2) the sponsor being in a position of higher power or greater influence, and (3) the sponsor directly advocating on behalf of the sponsored faculty member for “opportunities for skill development, high-visibility projects, access to decision-making forums” [26]. Sponsorship is a key driver of professional visibility and career mobility, particularly regarding advancement to leadership roles for women and for underrepresented minorities [27,28]. Prior research, however, has shown that women may not be identified for opportunities nor do they seek sponsorship as actively as men and tend to receive less sponsorship as they progress from early career to midcareer and beyond [26,29-31]. Self-advocacy may be one difference that lowers sponsorship-related opportunities for women. In our study, sponsorship disparities by track were also notable with TT faculty significantly more likely to serve as sponsors and to have received sponsorship themselves (sponsees), such as being nominated for national presentations, leadership programs, or publications. Given the inherent standardized structure for advancement on the TT, TT faculty may be more conditioned or socialized to the concept of sponsorship. Additionally, activities such as serving as coauthor, presenting at a conference, or being asked to serve as a peer reviewer may be more common scholarly activities for TT faculty compared to CT faculty and thus more accessible as sponsorship opportunities.

For structured sponsorship at OSUCOM, WIMS hosts sponsorship parties to allow both TT and CT faculty to make direct requests for specific sponsorship needs to a group of high-level leadership [32]. These sponsorship parties have been well received by both sponsees and sponsors. In addition, the feedback from these parties has been used to inform departments, centers, and units on how to incorporate sponsorship opportunities for all faculty. Furthermore, awareness of the importance of sponsorship alongside mentorship for career success is critical, and WIMS has worked to raise awareness of what sponsorship looks like in academic medicine, including through definitions provided in the survey.

#### Career Advancement Programming

Formal career development programs have reported success in faculty retention, higher rates of promotion, and transition into senior leadership roles [7,33,34]. This survey-based study also highlighted career advancement programming that can be implemented more broadly and that incorporates participation of both the TT and the CT. Such programming is synergistic and provides opportunities for collaboration that allow TT and CT faculty to learn from one another**.** In addition, programs are scalable to departments and other institutions.

In 2022, WIMS developed the Research Accelerator Program to Translate, Innovate and Commercialize (RAPTIC), which supports faculty researchers in both TT and CT roles to start a new clinical research portfolio or strengthen their existing research programs to grow toward more innovation-based research. RAPTIC encourages greater equity in the identification of intellectual property (IP) and product development toward commercialization and clinical implementation. The program is now being leveraged more broadly across all tracks and ranks in the academic medicine community across campus.

Historically, innovation and inventorship, IP protection, commercialization, and spin-out company formation spaces have been overrepresented by male faculty [35-37]. At the initial entry into RAPTIC, all faculty are provided a trained and compensated peer research coach to strengthen their research programs, while supporting them to keep an eye on innovation and ideation. Faculty can learn to identify and protect their potential IP. RAPTIC has uncovered that TT and CT faculty tend to have different barriers to identifying potential IP, which can range from identification of molecules that are potential drug targets (tenure) to digital health innovations or novel surgical implementations (clinical). At the completion of RAPTIC, formal career development programming has ensured equitable access to the IP/commercialization landscape for all faculty, broadening participation in innovation research by lowering the barriers to knowledge, resources, and mentors.

Grant funding is a key barrier identified by TT faculty. Given the current challenges in federal funding that did not exist at the time of our survey, diversifying funding sources is more important than ever for research-active faculty across all tracks. Innovation funding, including Small Business Technology Transfer (STTR) and Small Business Innovation Research (SBIR), industry codevelopment, or product development/innovation grant mechanisms at federal agencies are all promising alternatives to traditional funding sources, which could also bring IP revenue back to the university.

WIMS also led the development of the Big Ten Academic Alliance (BTAA) CommUNITYten Collaborative of Women in Medicine and Biomedical Sciences [38]. The goal of the collaborative was to provide opportunities for networking, gaining access for visiting lectures, obtaining external letters of evaluation for promotion, while also cultivating opportunities for mentorship, collaboration, and sponsorship. This collaborative consists of the 20 past, current, and future BTAA institutions. OSU hosted the inaugural conference in June 2024, drawing in over 180 participants from 9 BTAA institutions, 6 industry companies, staff, and trainees. In addition, the collaborative launched an environmental scan survey in October 2024 to understand career advancement barriers across the collaborative and learn about best practices BTAA institutions implement to advance gender equity. The CommUNITYten Collaborative continues to unite women-identifying faculty across academic tracks and ranks. Initiatives, such as BTAA-hosted webinar series on career and leadership advancement, allow BTAA institutions to share resources and promote networking among faculty across institutions.

Last, but not least, the literature shows that most interventions are aimed at early-career faculty, noting that mid- and late-career faculty, who often have different professional needs and career goals may require more individualized career guidance [7,23,33,34]. WIMS continues to develop career development programming to address the needs of all faculty, including those midcareer, caregivers, and those seeking leadership roles. WIMS is actively developing a Midcareer Seminar Series to support both TT and CT faculty during this vulnerable inflection point in their academic careers [39]. This program can be duplicated among institutions with career development programming that has proven successful at other AMCs [40-43]. WIMS is also developing career development programming to support OSUCOM alumni as they navigate new careers. These wraparound programs not only will provide scaffolding for career advancement for all but also are adaptable and scalable at other AMCs.

In summary, these findings highlight three possible institutional priority areas across appointment tracks:

(1) expanding structured mentorship in career guidance, particularly for CT and midcareer faculty; (2) implementing formal sponsorship pathways across tracks to enhance national visibility; and (3) instituting comprehensive cross-track career development programming in areas such as research and innovation acceleration

These priority areas offer practical implications with scalable impact to improve equity in academic medicine and to address persistent structural barriers this survey highlights.

### Limitations

This study is based on self-reported survey data from our single institution, which may limit generalizability. Formal validity testing was not performed. This survey was geared toward women-identifying faculty only, and the exclusion of male-identifying faculty precludes comparisons by gender identity. Conclusions may not truly reflect whether barriers were gender based or more broadly related to the academic track. Although virtually half of women-identifying faculty participated, the voluntary nature of the survey introduces the potential for nonresponse bias. It is possible that answers could have been affected by social desirability bias; however, the anonymous nature of the survey may have helped to mitigate this. This sample was also representative of the overall population of women-identifying faculty based on the proportions of respondents in each rank and track compared with the institutional demographic data from OSUCOM’s Office of Faculty Affairs; however, the voluntary nature of participation could introduce the possibility of self-selection bias, where those with stronger opinions were more likely to respond.

Not all questions required an answer, and there was attrition over the course of the survey, potentially impacting data collection. This survey did not explore informal mentorship relationships, which may positively impact academic progression. We did not ask questions on department culture or for reasons certain barriers exist, such as grant funding and administrative load, and thus career barriers may, in fact, be more nuanced. We did not ask how intersectional identities of gender, race, or ethnicity may play a role in career advancement by track, department, or specialty. Intersectionality would be an important area for future study.

This study was intentionally designed as a descriptive, exploratory cross-sectional analysis. Accordingly, the primary analyses were group comparisons presented as counts/percentages, with significance assessed using chi-square tests (with corresponding *P* values). Because multivariable modeling was not part of the prespecified analytic plan for this descriptive study, logistic regression was not conducted in the current manuscript, and odds ratios (ORs) with 95% CIs were not included. Similarly, effect size measures were not considered within the scope of this descriptive analysis; thus, statistically significant findings should be interpreted with caution. Other such survey variables could be evaluated as outcomes in logistic regression in a future, hypothesis-driven analysis; however, that would represent a different analytic objective and is beyond the scope of this descriptive paper.

Other topics for future research should include broader gender representation; exploring intersectionality of gender, race/ethnicity, track, rank, department, and specialty; examining ongoing longitudinal analyses of career trajectories by track; and measuring the impact of institutional interventions aimed at mitigating identified barriers. Further examination could determine career development programming that could be successfully leveraged nationally to address needs for both TT and CT faculty.

### Conclusion

Our findings highlight that TT and CT women-identifying faculty experience different career landscapes with distinct track-specific barriers to career advancement. CT women-identifying faculty are particularly vulnerable in areas such as mentorship and sponsorship, while TT women-identifying faculty experience more research-related challenges. With an increasing number of women being hired as CT faculty, it is critical for AMCs to increase and maintain support for both TT and CT faculty regarding mentorship, sponsorship, and career advancement. Given distinct track-specific barriers, AMCs must implement institutional-level strategies and targeted career development programming to address the unique challenges facing women across all academic tracks and ranks.

## Data Availability

The data generated and analyzed during this study are not publicly available due to concerns about maintaining participant confidentiality in a single-institution survey. Aggregate data findings are available from the corresponding author upon reasonable request.

## Appendix

Multimedia Appendix 1 WIMS survey questions. WIMS: Widening Impact in Medicine and Science (formerly Women in Medicine and Science).

Multimedia Appendix 2 Specific career advancement barriers by track among women-identifying faculty survey participants at the Ohio State University College of Medicine. Questions are presented in a “select all that apply" format, with multiple choices allowed.

Multimedia Appendix 3 High resolution version of Figure 1.

## Abbreviations

AMC: academic medical center
BTAA: Big Ten Academic Alliance
CT: clinical track
IP: intellectual property
OSU: Ohio State University
OSUCOM: The Ohio State University College of Medicine
RAPTIC: Research Accelerator Program to Translate, Innovate and Commercialize
TT: tenure track
WIMS: Widening Impact in Medicine and Science (formerly Women in Medicine and Science) Organization

## References

[1] Richter KP, Clark L, Wick JA, Cruvinel E, Durham D, Shaw P, Shih GH, Befort CA, Simari RD (2020). Women Physicians and Promotion in Academic Medicine. N Engl J Med.

[2] Jena AB, Khullar D, Ho O, Olenski AR, Blumenthal DM (2015). Sex differences in academic rank in US medical schools in 2014. JAMA.

[3] Nonnemaker L (2000). Women physicians in academic medicine: new insights from cohort studies. N Engl J Med.

[4] Lautenberger DM, Ma VM, Dandar MA (2024). The state of women in academic medicine 2023-2024: progressing toward equity. Association of American Medical Colleges.

[5] Franks A, Calamur N, Dobrian A, Danielsen M, Neumann SA, Cowan E, Weiler T (2022). Rank and tenure amongst faculty at academic medical centers: a study of more than 50 years of gender disparities. Acad Med.

[6] Mallon WT, Cox N (2024). Promotion and tenure policies and practices at U.S. medical schools: is tenure irrelevant or more relevant than ever?. Acad Med.

[7] Purkey NJ, Han P, Woodward A, Davis AS, Johnston L, Klein R, Krawczeski CD, Leeman KT, Machut KZ, Patel MD, Scala M, McBride ME (2025). Advancing women physicians in academic medicine: a scoping review. Acad Med.

[8] Xierali I, Nivet M, Syed Z, Shakil A, Schneider F (2021). Recent trends in faculty promotion in U.S. medical schools: implications for recruitment, retention, and diversity and inclusion. Acad Med.

[9] Office of Academic Affairs (2025). Procedures and guidelines handbook. The Ohio State University.

[10] (2025). Institutional research and planning: faculty trends and demographics. The Ohio State University.

[11] Liu M, Mallon WT (2004). Tenure in transition: trends in basic science faculty appointment policies at U.S. medical schools. Acad Med.

[12] Mullangi S, Blutt MJ, Ibrahim S (2020). Is it time to reimagine academic promotion and tenure?. JAMA Health Forum.

[13] Lufler RS, McNulty MA (2022). The glass ceiling thickens: the impact of COVID-19 on academic medicine faculty in the United States. Med Educ Online.

[14] Walling A, Nilsen K (2018). Tenure appointments for faculty of clinical departments at U.S. medical schools: does specialty designation make a difference?. Acad Med.

[15] Jeffe D, Yan Y, Andriole D (2019). Competing risks analysis of promotion and attrition in academic medicine: a national study of U.S. medical school graduates. Acad Med.

[16] Braxton MM, Infante Linares JL, Tumin D, Campbell KM (2020). Scholarly productivity of faculty in primary care roles related to tenure versus non-tenure tracks. BMC Med Educ.

[17] Pololi LH, Evans AT, Gibbs BK, Krupat E, Brennan RT, Civian JT (2013). The experience of minority faculty who are underrepresented in medicine, at 26 representative U.S. medical schools. Acad Med.

[18] Campbell KM, Rodríguez JE, Brownstein NC, Fisher ZE (2017). Status of tenure among black and Latino faculty in academic medicine. J Racial Ethn Health Disparities.

[19] Xierali I, Nivet M, Syed Z, Shakil A, Schneider F (2020). Trends in tenure status in academic family medicine, 1977-2017: implications for recruitment, retention, and the academic mission. Acad Med.

[20] Farkas AH, Bonifacino E, Turner R, Tilstra SA, Corbelli JA (2019). Mentorship of women in academic medicine: a systematic review. J Gen Intern Med.

[21] Sambunjak D, Straus SE, Marusić Ana (2006). Mentoring in academic medicine: a systematic review. JAMA.

[22] Files JA, Blair JE, Mayer AP, Ko MG (2008). Facilitated peer mentorship: a pilot program for academic advancement of female medical faculty. J Womens Health (Larchmt).

[23] Bellini L, Kaplan B, Fischel J, Meltzer C, Peterson P, Sonnino R (2020). The definition of faculty must evolve: a call to action. Acad Med.

[24] Mousa M, Boyle J, Skouteris H, Mullins AK, Currie G, Riach K, Teede HJ (2021). Advancing women in healthcare leadership: a systematic review and meta-synthesis of multi-sector evidence on organisational interventions. EClinicalMedicine.

[25] (2025). Faculty mentoring program with mentorcliQ. The Ohio State University.

[26] Schwartz R, Williams MF, Feldman MD (2024). Does sponsorship promote equity in career advancement in academic medicine? A scoping review. J Gen Intern Med.

[27] Ibarra H, Carter NM, Silva C (2010). Why men still get more promotions than women. Harv Bus Rev.

[28] Ayyala MS, Skarupski K, Bodurtha JN, González-Fernández M, Ishii LE, Fivush B, Levine RB (2019). Mentorship is not enough: exploring sponsorship and its role in career advancement in academic medicine. Acad Med.

[29] Levine RB, Ayyala MS, Skarupski KA, Bodurtha JN, Fernández MG, Ishii LE, Fivush B (2021). "It's a little different for men"-sponsorship and gender in academic medicine: a qualitative study. J Gen Intern Med.

[30] Williams MF, Yank V, O'Sullivan P, Alldredge B, Feldman MD (2023). Faculty knowledge, actions, and perceptions of sponsorship: an institutional survey study. Med Educ Online.

[31] Mahendran GN, Walker ER, Bennett M, Chen AY (2022). Suturing the gender gap through sponsorship: the role of sponsorship in female entry and advancement through their surgical careers. Am J Surg.

[32] Silver JK (2023). Six practical strategies to mentor and sponsor women in academic medicine. J Med Internet Res.

[33] Nowling TK, McClure E, Simpson A, Sheidow AJ, Shaw D, Feghali-Bostwick C (2018). A focused career development program for women faculty at an academic medical center. J Womens Health (Larchmt).

[34] Chang S, Guindani M, Morahan P, Magrane D, Newbill S, Helitzer D (2020). Increasing promotion of women faculty in academic medicine: impact of national career development programs. J Womens Health (Larchmt).

[35] Schuster WM, Marcowitz-Bitton M, Gerhardt DR (2022). The gender gap in academic patenting. University of North Carolina School of Law.

[36] Ding WW, Murray F, Stuart TE (2006). Gender differences in patenting in the academic life sciences. Science.

[37] Aneja A, Reshef O, Subramani G (2024). Attrition and the gender patenting gap. Rev Econ Stat.

[38] Iyer MS, Moe A, Massick S, Davis J, Ballinger M, Townsend K (2025). Development of the Big Ten Academic Alliance Collaborative for Women in Medicine and Biomedical Science: "we built the airplane while flying it". JMIR Form Res.

[39] Paradis KC, Kerr EA, Griffith KA, Cutter CM, Feldman EL, Singer K, Spector ND, Ubel PA, Jagsi R (2024). Burnout among mid-career academic medical faculty. JAMA Netw Open.

[40] Lewiss RE, Silver JK, Bernstein CA, Mills AM, Overholser B, Spector ND (2020). Is academic medicine making mid-career women physicians invisible?. J Womens Health (Larchmt).

[41] Chaudron LH, Anson E, Bryson Tolbert JM, Inoue S, Cerulli C (2021). Meeting the needs of mid-career women in academic medicine: one model career development program. J Womens Health (Larchmt).

[42] Campion MW, Bhasin RM, Beaudette DJ, Shann MH, Benjamin EJ (2016). Mid-career faculty development in academic medicine: how does it impact faculty and institutional vitality?. J Fac Dev.

[43] Mid-career faculty leadership development seminar. Association of American Medical Colleges.

