# Supplementary material for: Career Advancement Challenges for Women in Tenure Versus Clinical Tracks in Academic Medicine: Cross-Sectional Survey Study
Source: JMIR Form Res. 2026 May 29;10:e83374. doi: 10.2196/83374 (PMC13263658; doi:10.2196/83374)
Supplement: Multimedia Appendix 1 [file formative_v10i1e83374_app1.doc]

# **Appendix 1: WIMS Advocacy Women Faculty Survey**

# **DEMOGRAPHICS QUESTIONS**

# **Q:** Are you woman-identifying (Woman+)?

# Yes

# No

# The survey conditionally closes if (No, you are not women-identifying (Woman+).)

# The following questions are conditionally shown if: (Yes, you are woman-identifying (Woman+).)

# **Q:** What is your Promotion Track?

- Clinical Track
- Research Track
- Tenure Track
- Associated Track

# **Q:** What is your Clinical Track Pathway?

- Clinical Educator
- Clinical Excellence
- Clinical Scholar
- Unsure

**Q:** What is your Associated Track?

- Adjunct
- Emeritus
- Practice
- Lecturer
- Returning Retiree
- Returning Retiree AND Emeritus
- Tenure Track <50% FTE
- Visiting
- Unsure

**Q:** What is your Current Rank?

- Instructor
- Assistant Professor
- Associate Professor
- Professor
- N/A

**Q:** Years in Current Rank:

- 0-5 years
- 6-10
- 11+

**Q:** Your Primary Department/Tenure Initiating Unit (optional)

Drop-down menu listing all departments at the COM.

| **Anesthesiology** |
| --- |
| **Biological Chemistry and Pharmacology** |
| **Biomedical Education and Anatomy** |
| **Dermatology** |
| **Emergency Medicine** |
| **Family and Community Medicine** |
| **Health and Rehabilitation Sciences, School of** |
| **Internal Medicine** |
| **Microbial Infection and Immunity** |
| **Neurological Surgery** |
| **Neurology** |
| **Neuroscience** |
| **Obstetrics & Gynecology** |
| **Ophthalmology and Visual Sciences** |
| **Orthopedics** |
| **Otolaryngology** |
| **Pathology** |
| **Pediatrics** |
| **Physical Medicine & Rehabilitation** |
| **Physiology and Cell Biology** |
| **Plastic & Reconstructive Surgery** |
| **Psychiatry & Behavioral Health** |
| **Radiation Oncology** |
| **Radiology** |
| **Surgery** |
| **Urology** |

**Q:** Your Primary Division

- - Enter the Name of your Primary Division _____________________________
  - Prefer Not to Answer
  - N/A

**Q:** Which race best describes you? (Please choose only one.)

- - American Indian or Alaska Native
  - Asian
  - Black or African American
  - Native Hawaiian or Other Pacific Islander
  - White
  - Multiracial
  - Other
  - Prefer not to answer

**Q:** What ethnicity best describes you?

- Hispanic/Latino
- Non-Hispanic/Latino
- Prefer not to answer

**MENTORSHIP**

**Q:** Do you currently have a mentor?

- - Yes
  - No

# The following question is conditionally shown if: (No, you do not currently have a mentor.)

# List the reasons you do not have a mentor: ________________________

The following questions are conditionally shown if: (Yes, you do currently have a mentor).

**Q:** Is/are your mentor(s): (Check all that apply):

- From your department/division
- From another department/division at OSUCOM/Nationwide Children’s Hospital (NCH)
- External to OSUCOM/NCH

# **Q:** Considering your most meaningful mentorship relationship, is your mentor:

- Assigned to you
- Self-initiated

# **Q:** Considering your most meaningful mentorship relationship, what is/was the primary focus?

- Administrative
- Career Development
- Clinical Service
- Promotion & Tenure
- Research/Scholarship
- Teaching
- Work/Life balance
- Other, please specify: ________________________________________

# **Q:** Considering your most meaningful mentorship relationship, how frequently have you met with this mentor in the past 12 months?

- 0
- 1-2 times
- >3 times
- Other, please specify: ________________________________________

# **Q:** In which of the following areas would you like more mentorship? (Check all that apply).

- Collaboration—initiating and maintaining
- Communicating with individuals with different learning styles
- Educating trainees
- Grant Writing
- Leadership skills
- Multidisciplinary teamwork
- Negotiation skills
- Understanding and leveraging available resources in the COM/Medical Center
- Work/Life balance
- Other, please specify: _______________________________________________

# **Q:** Do you serve as a mentor in your department?

# Yes

# No

# The following questions are conditionally shown if: (Yes, you do serve as a mentor in my department.)

**Q:** How many of your mentees are:

- From your department? __
- From another department within OSU? __
- External to OSU? __
- Assigned to you? __
- Initiated by the mentee? __

# The following question is conditionally shown if: (No, you do not serve as a mentor in my department.)

**Q:** Why are you not currently serving as a mentor?:

- I would like to but do not have the time.
- I would like to but have not been asked.
- I would like to but have not identified a mentee.
- I do not want to serve as a mentor at this time.
- Other, please elaborate: ___________________________________________

# **PROMOTION AND TENURE**

**Q.** Do you understand the Appointment, Promotion, & Tenure (AP&T) process and the requirements for promotion?

- Yes, I know what I need to do.
- Yes, I understand most portions but could beneﬁt from clariﬁcations/additional detail on other portions.
- No, I am not aware of the requirements/I do not understand the AP&T requirements.

# **Q.** Have you had the opportunity to review your department’s AP&T document?

- Yes, I have access to the AP&T document and understand the process.
- Yes, I have access to the AP&T document, but I have not reviewed it.
- No, I don’t know where to look for the document and/or have not received any information on what can be done for promotion.

# **Q:** Has your chair discussed your path to promotion during your annual review?

- Yes, it was discussed, and I have an understanding of my current path to promotion.
- Yes, it was brought up but not discussed in detail.
- No, it was not discussed at my annual review.
- I have not had an annual review.

# **Q:** Have you utilized dossier coach/reviewer services?

- Yes
- No, because these services are not available in my department.
- No, because I am/was not aware of these services.
- No, because I did not need these services.

# **Q:** What level of administrative support is available in your department to help with Promotion & Tenure?

- Dedicated P&T coordinator
- General administrative support
- I am not aware of any administrative support for promotion in my department.
- I am not sure.

**Q:** What, if any, barriers to career advancement have impacted your career path? (Select all that apply)

- Burnout
- Caretaking responsibilities (Childcare, Elder care, etc.)
- Financial hardships
- Impact of COVID pandemic
- Increased administrative responsibilities
- Increased patient load
- Lack of administrative support
- Lack of funding
- Lack of institutional support
- Lack of mentorship
- Lack of sponsorship
- Productivity requirements
- Self-care/illness
- Unequal distribution of teaching responsibilities
- I do not feel that any barriers have impacted my career path
- Other, please elaborate: ___________________________________________

**SPONSORSHIP**

A sponsor is someone who advocates and identiﬁes you for a career-advancing opportunity, such as a nomination for a leadership role, award, and/or speaking engagement. This can be for a single occurrence or for multiple opportunities.

**Q:** In your current faculty position have you been sponsored by another individual?

- - Yes
  - No

# The following question is conditionally shown if: (No, you have not been sponsored by another individual.)

Q: Is there a reason you have not received sponsorship?

- Have not yet been sponsored
- Have not yet identiﬁed potential sponsors
- Opportunities go to others
- Had to decline
- Other, please specify: ________________________

# The following questions are conditionally shown if: (Yes, you have been sponsored by another individual.)

# **Q:** For which of the following opportunities have you been sponsored? (Select all that apply.)

- Editorial Board
- Grand Rounds
- Grant reviewer
- Institutional Committee Work
- Institutional Search Committee(s)
- Leadership development programs
- National conference panelist/moderator
- National Conference Presentation(s)
- National society committee nominations
- Nomination for awards/recognition
- Nomination to mission-critical or high visibility roles in division/department
- Publications, 1st/senior authorship
- Social media recognition
- Other, please specify: ____________________________

# **Q.** Do you feel that you have adequate sponsorship from senior faculty at OSU to achieve national recognition?

- Yes
- No

# The following question is conditionally shown if: (No, I do not feel I have adequate sponsorship from senior faculty at OSU to achieve national recognition.)

**Q:** If you have not received adequate sponsorship, check all that apply:

- Have not yet been sponsored
- Have not yet identiﬁed potential sponsors
- Opportunities go to others
- Had to decline
- Other, please specify: ________________________

# Q. In your current position, have you sponsored an OSU faculty member?

# Yes

# No

# The following question is conditionally shown if: (Yes, in your current position, you have sponsored an OSU faculty member.)

**Q:** For which of the following, have you sponsored an OSU faculty member? (Check all that apply)

- Editorial Board
- Grand Rounds
- Grant reviewer
- Institutional Committee Work
- Institutional Search Committee(s)
- Leadership development programs
- National conference panelist/moderator
- National Conference Presentation(s)
- National society committee nominations
- Nomination for awards/recognition
- Nomination to mission-critical or high visibility roles in division/department
- Publications, 1st/senior authorship
- Social media recognition
- Other, please specify: ____________________________

# The following question is conditionally shown if: (No, in your current position, you have not sponsored an OSU faculty member.)

**Q.** If you have not been a sponsor, then why not? Please elaborate: ______________________

**Q.** How can WIMS help you in your career development and in faculty support? Please comment on current programming or suggested options. ________________________________

**Q.** Is there any additional information that you wish to share with WIMS?
