# Supplementary material for: Career Advancement Challenges for Women in Tenure Versus Clinical Tracks in Academic Medicine: Cross-Sectional Survey Study
Source: JMIR Form Res. 2026 May 29;10:e83374. doi: 10.2196/83374 (PMC13263658; doi:10.2196/83374)
Supplement: Multimedia Appendix 2 [file formative_v10i1e83374_app2.docx]

**Appendix 2. Specific career advancement barriers by track among women-identifying faculty survey participants at OSUCOM. Question presented as a “select all that apply format” with multiple choice allowed.**

| **Variable** | **Level** | **Tenure Track (n=101)** | **Clinical Track (n=391)** | **Total (n=492)** | **P-value** |
| --- | --- | --- | --- | --- | --- |
| Burnout | Yes | 48 (47.52%) | 187 (47.83%) | 235 (47.76%) | 0.9569 |
|  | No | 53 (52.48%) | 204 (52.17%) | 257 (52.24%) |  |
| Caretaking responsibilities (Child care, Elder care, etc.) | Yes | 54 (53.47%) | 217 (55.5%) | 271 (55.08%) | 0.7142 |
|  | No | 47 (46.53%) | 174 (44.5%) | 221 (44.92%) |  |
| Financial hardships | Yes | 9 (8.91%) | 10 (2.56%) | 19 (3.86%) | 0.0031 |
|  | No | 92 (91.09%) | 381 (97.44%) | 473 (96.14%) |  |
| I do not feel that any barriers have impacted my career path | Yes | 8 (7.92%) | 28 (7.16%) | 36 (7.32%) | 0.7938 |
|  | No | 93 (92.08%) | 363 (92.84%) | 456 (92.68%) |  |
| Impact of COVID pandemic | Yes | 50 (49.5%) | 75 (19.18%) | 125 (25.41%) | <.0001 |
|  | No | 51 (50.5%) | 316 (80.82%) | 367 (74.59%) |  |
| Increased administrative responsibilities | Yes | 47 (46.53%) | 160 (40.92%) | 207 (42.07%) | 0.7222 |
|  | No | 54 (53.47%) | 231 (59.08%) | 285 (57.93%) |  |
| Increased patient load | Yes | 10 (9.9%) | 187 (47.83%) | 197 (40.04%) | <.0001 |
|  | No | 91 (90.1%) | 204 (52.17%) | 295 (59.96%) |  |
| Lack of administrative support | Yes | 39 (38.61%) | 170 (43.48%) | 209 (42.48%) | 0.3780 |
|  | No | 62 (61.39%) | 221 (56.52%) | 283 (57.52%) |  |
| Lack of funding | Yes | 28 (27.72%) | 66 (16.88%) | 94 (19.11%) | 0.0135 |
|  | No | 73 (72.28%) | 325 (83.12%) | 398 (80.89%) |  |
| Lack of institutional support | Yes | 31 (30.69%) | 103 (26.34%) | 134 (27.24%) | 0.3813 |
|  | No | 70 (69.31%) | 288 (73.66%) | 358 (72.76%) |  |
| Lack of mentorship | Yes | 25 (24.75%) | 134 (34.27%) | 159 (32.32%) | 0.0682 |
|  | No | 76 (75.25%) | 257 (65.73%) | 333 (67.68%) |  |
| Lack of sponsorship | Yes | 23 (22.77%) | 62 (15.86%) | 85 (17.28%) | 0.1012 |
|  | No | 78 (77.23%) | 329 (84.14%) | 407 (82.72%) |  |
| Other | Yes | 20 (19.8%) | 43 (11%) | 63 (12.8%) | 0.0182 |
|  | No | 81 (80.2%) | 348 (89%) | 429 (87.2%) |  |
| Productivity requirements | Yes | 19 (18.81%) | 150 (38.36%) | 169 (34.35%) | 0.0002 |
|  | No | 82 (81.19%) | 241 (61.64%) | 323 (65.65%) |  |
| Self-care/Illness | Yes | 11 (10.89%) | 58 (14.83%) | 69 (14.02%) | 0.3090 |
|  | No | 90 (89.11%) | 333 (85.17%) | 423 (85.98%) |  |
| Unequal distribution of teaching responsibilities | Yes | 7 (6.93%) | 55 (14.07%) | 62 (12.6%) | 0.0541 |
|  | No | 94 (93.07%) | 336 (85.93%) | 430 (87.4%) |  |
