# Supplementary figures and images for: Career Advancement Challenges for Women in Tenure Versus Clinical Tracks in Academic Medicine: Cross-Sectional Survey Study
Source: JMIR Form Res. 2026 May 29;10:e83374. doi: 10.2196/83374 (PMC13263658; doi:10.2196/83374)

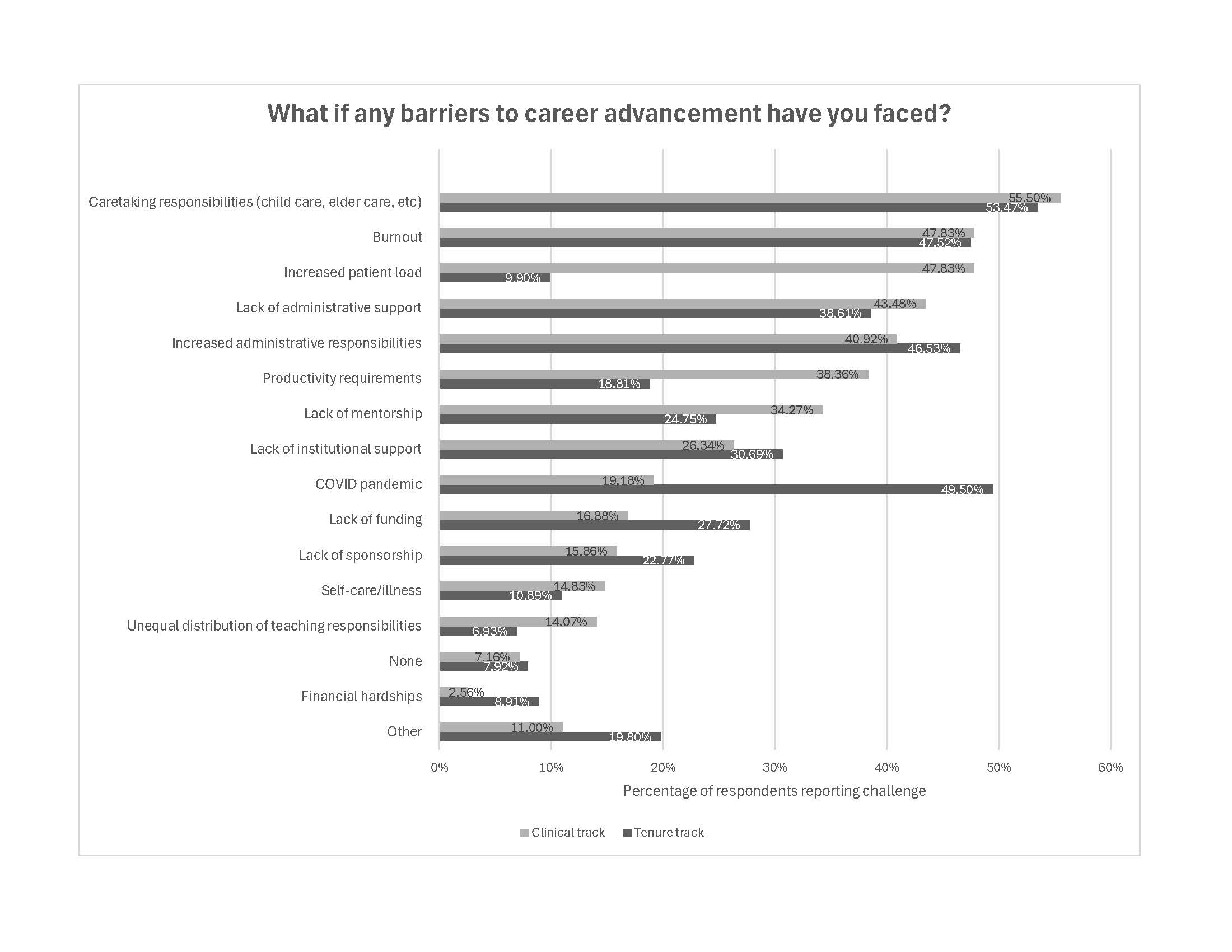

Supplement: Multimedia Appendix 3 [file formative_v10i1e83374_app3.png]
